# Clinical Factors and Outcomes Associated With Community Treatment Order: A Service Evaluation

**DOI:** 10.1192/bjo.2026.11551

**Published:** 2026-06-30

**Authors:** Natalie Cook, Nilamadhab Kar

**Affiliations:** 1 Black Country Healthcare NHS Foundation Trust, Wolverhampton, United Kingdom; 2 University of Wolverhampton, Wolverhampton, United Kingdom

## Abstract

**Aims:**

We intended to study the clinical outcomes and risk factors associated with patients on Community Treatment Order (CTO) and those discharged from Section 3 (S3).

**Methods:**

The sample included adult patients admitted (n: 123) on Section 3 of the Mental Health Act in 2022; 11 of them are currently on CTO, along with a further 55 patients currently on CTO, suggesting the sample of 66 in CTO and 112 in the S3 group. Historical and previous 12-month status of clinical and risk factors, readmission rates, duration to first readmission, and total inpatient days were collected from medical records.

**Results:**

Sample (n: 178) consisted of 106 (59.6%) male and 72 (40.4%) female patients; with 52.8% having Caucasian ethnicity; males made up 52.1% of the Caucasian group, vs.69.2% in BME patients (P<0.05). The proportion of men was 69.7% on CTO and 53.6% on S3 (P<.05). The mean age of the groups (CTO: 46.1 ± 17.2; S3: 45.5 ± 17.4) was comparable, and there were no differences in ethnicity, employment, and accommodation status between the two groups.

The number of diagnoses and comorbidities was comparable, whereas the presence of severe mental illness was significantly higher in the CTO: 97.0% vs. S3: 81.3% (p<0.005). History of risk to self, others, neglect, arrests, custodial sentence, probation, and being on CTO or s37/41 was comparable; forensic history and convictions were significantly more in CTO.

Duration from index discharge to readmission was considerably longer in CTO (439.2 ± 471.4) vs S3 (265.5 ± 281.2), which approached statistical significance (p: 0.059). The duration of first readmission and the nature of readmission (informal or formal) were comparable between the groups. Significantly more patients in CTO had depot and oral antipsychotics, and fewer had antidepressants compared to S3; whereas prescriptions of high-dose antipsychotics, more than one antipsychotic, mood stabilisers, and hypnotic drugs were comparable.

In the previous 12-month period, risk to self, to others, self-neglect, opiate use, psychotic symptoms, impaired or absent insight, poor engagement, and poor concordance were all significantly higher in CTO patients compared to those discharged from S3.

**Conclusion:**

There were considerable differences in a few areas of the CTO and S3 patient profile, e.g. forensic history, past convictions, and treatment with depot and antipsychotics. There were many clinical and risk-related factors in the previous 12 months, validating continuation of CTO in a proportion of patients. Future studies should consider differences in symptomatic presentations, functioning and patient-reported outcomes.

